# High dose intravenous vitamin c treatment in a patient with lung cancer: A case report

**DOI:** 10.15761/ccrr.1000243

**Published:** 2016-06-18

**Authors:** Michael J. González, Miguel J. Berdiel, Jorge R. Miranda- Massari, Desireé López, Joshua L. Rodríguez-López, Pedro A. Adrover-López, Jorge Duconge

**Affiliations:** 1 School of public health, Medical Sciences Campus, University of Puerto Rico, Ponce PR; 2 School of Pharmacy, Medical Sciences Campus, University of Puerto Rico, Ponce PR; 3 San Juan PR, Berdiel Clinic, Ponce PR; 4 School of Medicine, Ponce Health Sciences University, Ponce PR

**Keywords:** pulmonary cancer, lung cancer, vitamin C, ascorbic acid

## Abstract

Lung cancer is one of the most common cancers in both men and women. According to Dr. Abram Hoffer, patients with a better nutritional plan and daily Vitamin C supplementation improved their life quality. In this case report we present the case of a 56-year-old Hispanic male patient diagnosed with lung cancer on 2012. After undergoing surgery and chemotherapy, he started a high dose intravenous Vitamin C protocol on December 2013. The treatment continued until June 2015, when the patient decided to stop the treatment. A maximum of 75 gr of Vitamin C in 1,000 cc lactated Ringer’s was given three times a week in a period over a year and a half. Patient’s CEA levels continued to be within normal levels while high doses of Vitamin C infusions were given. Many case reports suggest that patients with lung cancer that received high doses of intravenous Vitamin C can live up to 10 years. A level of Vitamin C in plasma above 400 mg/dL is toxic to tumor cells, this can be achieved with periodic Vitamin C infusions. Our case support that the usage of high doses of IV Vitamin C can be effective in the treatment of patients with cancer without secondary effects.

## Introduction

Lung cancer is considered the second most common cancer in both men and women in the United States [1]. This type of cancer is more common in men but, the numbers are still increasing among women. According to the National Cancer Institute it is estimated that in 2015 lung cancer will account for approximately 26.8% of all cancer deaths in Americans. Based on the data of 2005 to 2011 from SEER the survival rate of lung cancer is 17.4% for 5 years. Tobacco smoking is considered the most common cause of lung cancer followed by exposure to asbestos and other chemicals [2]. Lung cancer risk is increased even with the exposure of second hand smoke.

There are many studies that show the effectiveness of intravenous vitamin C treatment in lung cancer and many other types of cancers as well. According to Dr. Abram Hoffer’s studies, patients with cancer that have improved their life quality are the ones that improve the quality of their diet and add daily vitamin C supplementation. He also found that the patients that were committed with the nutrition and supplementation program for a longer period of time; live longer than the patients who do not follow the program [3].

## Case report

A case of a 56 year old male Hispanic patient with a medical history of chronic smoking and associated COPD (chronic obstructive pulmonary disease) diagnosed with lung cancer since 2012. He underwent a left lower lobe segment and tumor resection in summer 2013. The patient also received chemotherapy with last cycle on November 2013. He also began adjuvant therapy with docetaxel and carboplatin by week 7 post-operative and completed this treatment in week 20 post-operative. No radiation therapy was offered.

The patient was started on high dose intravenous vitamin C protocol in December 2013 and continued until June 2015. Initially the patient was given a G6PD test. After showing no problem handling the IV vitamin C, the IV vitamin C protocol was implemented. The first dose of IV vitamin C infusion was 25 gr in 250 cc lactated Ringer’s during a 1hour infusion. The second infusion was 50 gr in 500cc of lactated Ringer’s over a period of 1.5 hour. The third infusion was 75 gr in 1,000 cc lactated Ringer’s for 2 hours. A maximum of 75 gr of vitamin C in 1,000 cc lactated Ringer’s was given three times a week in a little over 1.5 years period.

A series of laboratory values was performed in this patient throughout this treatment. On November 2012, the patient presented an elevated level of CEA in 6.5 ng/mL, aspartate aminotransferase (AST) in 44 u/L, and alanine aminotransferase (ALT) in77 u/L. A CT scan of the chest was also performed and showed calcifications of the coronary arteries and two nodules in the left mid lung and the other in the right upper lobe.

On March 2013, the levels of alanine aminotransferase (ALT) and CEA continue to be elevated in 67 u/L and 13 ng/mL, respectively. All other laboratories values were within normal levels. On August 2013, the biochemistry data showed an improvement in CEA levels to 1.9 ng/mg considered to be within normal levels while given high doses of vitamin C infusions. On November 2013, a PET/CT SCAN of the whole body was performed and found no areas of abnormal increase F 18 -FDG uptake to suggest a hypermetabolic lesion.

At this moment, the patient is still alive and enjoying activities of daily living. He decided to stop the IV vitamin C treatment.

## Discussion

The patient showed improvement while receiving high dose IV vitamin C infusions for 1.5 years and adjuvant therapy for just 27 weeks after the surgery on 2013. Laboratory values where within normal levels except for CEA, AST, and ALT. Some studies with high doses vitamin C have proven to be very effective against lung cancer [3,4]. This treatment has given the opportunity to many patients to increase their survival time and their quality of life. There are case reports describing patients with lung cancer, who began to receive high doses of IV vitamin C, living up to 10 years after diagnosis [3,4]. On the other hand, there is a case report of a patient diagnosed with adenocarcinoma of his right kidney with metastatic lesions in the liver and lung, received IV vitamin C treatment and after fifteen months of initial therapy the patient had no signs of progressive cancer and remained cancer free for 14 years [5]. There are studies that show that vitamin C acts as a potential chemotherapeutic agent [3–5]. Compared with some chemotherapeutic drugs that cause severe adverse side effects, vitamin C provides side benefits such as enhancing immune function and increasing collagen production [4]. It has been reported that a vitamin C plasma level above 400 mg/dL is toxic to tumor cells [4]. This concentration of vitamin C can be achieved in humans using IV vitamin C infusions periodically. Our data supports that high dose of IV vitamin C can be used in the management of patients with cancer.
